# From trunk and hip muscle function to class allocation: understanding the functional basis of wheelchair basketball classification

**DOI:** 10.3389/fspor.2026.1872207

**Published:** 2026-07-17

**Authors:** Karina Santos Guedes de Sá, José Irineu Gorla, Elisa Digo, Marie Lund Ohlsson, Anna Bjerkefors, Yves Vanlandewijck

**Affiliations:** 1 Department of Adapted Physical Activity, State University of Campinas (UNICAMP), Campinas, Brazil; 2 Graduate Program in Physical Exercise for Health Promotion, North Paraná University (UNOPAR), Londrina, Brazil; 3 Department of Mechanical and Aerospace Engineering, Politecnico di Torino, Turin, Italy; 4 Department of Physiology, Nutrition and Biomechanics, The Swedish School of Sport and Health Sciences (GIH), Stockholm, Sweden; 5 Department of Health Sciences, The Swedish Winter Sports Research Centre, Mid Sweden University, Östersund, Sweden; 6 Department of Neuroscience, Karolinska Institute, Stockholm, Sweden

**Keywords:** classification, functional assessment, muscle strength, trunk function, volume of action, wheelchair basketball

## Abstract

**Background:**

Functional assessment in wheelchair basketball (WB) has three evaluation stages: analysis of isolated functions, combined functional measures, and the observation of volume of action (VoA) in a sporting context. Sport class allocation relies on VoA assessed in controlled conditions (VoA-capacity), which corresponds to combined functional measures, and during game observation (VoA-performance). However, the contribution of each assessment phase to sport class allocation remains insufficiently understood. This study examines how trunk and hip muscle strength influences VoA-capacity and evaluates the extent to which VoA-capacity is associated with sport class. Additionally, it compares trunk muscle strength and VoA-capacity across sport classes to characterize functional differences between groups.

**Methods:**

A cross-sectional observational study was conducted with 40 officially classified WB players (sport classes 1.0–4.5). Trunk and hip muscle strength were assessed using the Manual Muscle Test (MMT). VoA-capacity was calculated as the sum of maximal trunk reaches in six directions (flexion, extension, rotation for both sides and lateral flexion for both sides) using an Inertial Measurement Unit (IMU) positioned at C7 (seventh cervical vertebra). Multiple linear regression, ordinal regression, Kruskal–Wallis, and Dunn's *post-hoc* tests were applied.

**Results:**

Total Trunk-Hip muscle strength significantly predicted VoA-capacity (*p* < 0.001), explaining 80% of its variance (*R*^2^ = 0.80). When analyzed separately, both trunk strength (*p* < 0.001) and hip strength (*p* = 0.006,) were significantly associated of VoA-capacity. VoA-capacity was a significant predictor of sport class (*p* < 0.001). Significant differences were found between sport classes for VoA-capacity (*p* < 0.001) and for total Trunk-Hip muscle strength (*p* < 0.001), with *post-hoc* comparisons showing the largest contrasts between lower (1.0–2.0) and higher (4.0–4.5) sport classes.

**Conclusion:**

Trunk and hip muscle strength showed associations with VoA capacity, suggesting their relevance for understanding players’ ability to perform dynamic seated movements. VoA-capacity, was also associated with sport class allocation. Although muscle strength is not a classification criterion in WB, profiling players’ muscular strength may offer complementary insights in complex classification scenarios. These findings provide preliminary functional evidence of WB classification and support future investigations integrating VoA-performance to refine assessment models.

## 1 Introduction

**Figure 1:**
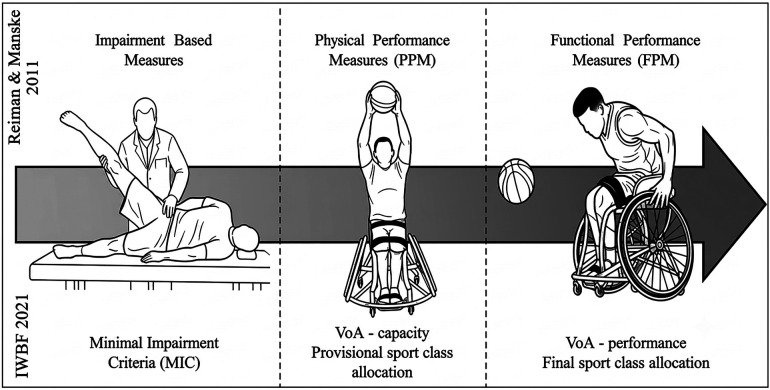
Functional evaluation flow.

According to Reiman & Manske (1), the functional assessment, is the process of assessing an individual's ability to perform movements and tasks that replicate the functional demands of daily life, sports, or occupational activities. The functional process is progressing through three stages: I - impairment-based measures, which assess isolated functions; II - physical performance measures (PPM), which integrate functions in specific functional tasks; and III - functional performance measures (FPM), which analyze integrated functions in sports activities (1). The International Wheelchair Basketball Federation (IWBF) adopts a similar approach, although using a different terminology (see Figure 1): I - verification of minimum impairment criteria (MIC) to check the player's eligibility; II - assessment of the volume of action (VoA) here referred to as VoA-capacity, to determine the player's provisional sport class by evaluating the impact of the eligible impairment on the capacity to perform wheelchair basketball-specific activities under standardized assessment conditions; and III - observation of VoA during the game, here referred to as VoA-performance, to determine the player's final sport class by evaluating how the eligible impairment affects the execution of sport-specific activities in the competition environment (2).

The allocation of sport classes in Wheelchair Basketball (WB) is based on the player's VoA, defined as the limit to which the player can voluntarily move the trunk in any direction and return to the seated upright position without external support, holding the ball with both hands (2). Following the functional assessment model of WB, there are two types of VoA: VoA-capacity, evaluated in standardized and controlled tests, which represents the player's movement potential; and VoA-performance, observed during the game, reflecting the use of this VoA during realistic multi-task WB-actions (2). The VoA measures determine the functional profiles of the main WB sport classes (1.0, 2.0, 3.0, 4.0 and 4.5), with each profile representing the characteristic impact of the eligible impairment on wheelchair basketball-specific activities. Players in the intermediate sport classes (1.5, 2.5 and 3.5) present hybrid functional characteristics, and therefore do not demonstrate a specific functional profile (2). The allocation to sport classes is based on the VoA. VoA synthesizes the player's functional capacity and serves as the foundation for sport class allocation. WB-sport class profiles are presented in Table 1.

**Table 1:** Class profiles in WB.

Class/trunk movement	Sagittal plane	Horizontal plane	Frontal plane
1.0	Has little or no active movement	No active movement	No active movement
2.0	Has upper trunk flexion (approximately 45° of flexion)	Has upper trunk rotation	No active movement
3.0	Has full trunk flexion (trunk as a unit)	Has full trunk rotation (trunk as a unit)	No active movement
4.0	Has full trunk flexion (trunk as a unit)	Has full trunk rotation (trunk as a unit)	Has full trunk lateral flexion to one side only
4.5	Has full trunk flexion (trunk as a unit)	Has full trunk rotation (trunk as a unit)	Has full trunk lateral flexion to both sides

In WB, athletes may be eligible for sport class evaluation if they present an eligible impairment that affects lower-limb function and, consequently, sport performance (2). These impairments include impaired muscle power, impaired passive range of movement, limb deficiency, leg length difference, impairment of motor coordination (hypertonia, ataxia, and athetosis), as defined by the International Paralympic Committee and adopted by the IWBF (2). Although different eligible impairments may be present, trunk function is a key determinant of sport class because it directly influences wheelchair propulsion, balance, reaching, ball handling, and other wheelchair basketball-specific activities (2).

Even though the trunk plays a fundamental role in the classification of WB-players, according to the current IWBF Classification Manual, trunk muscle strength is not directly assessed as an isolated impairment measure during the classification process (2). Instead, the evaluation of muscle strength is limited to hip and lower-limb function for determining the MIC, whereas sport class allocation is based on the athlete's VoA (2). Since VoA aims to capture the functional impact of trunk impairment on wheelchair basketball-specific activities, trunk muscle strength and range of motion are assessed indirectly through the observation of trunk movement execution during standardized testing. Specifically, classifiers infer trunk muscle strength from the athlete's ability to actively initiate, control, and sustain trunk movements against gravity and while performing standardized functional tasks. Consequently, the current classification system does not explicitly quantify trunk muscle strength, despite its potential contribution to trunk control and sport-specific performance.

Santos et al. (3), compared trunk isometric strength and stability limits across different sport classes in WB players and found that players from higher sport classes exhibited greater isometric strength and better trunk balance. Complementarily, Marszalek and Molik (4) investigated the maximum range of active trunk movement in WB players and identified significant differences between adjacent sport classes in the three planes of movement highlighting trunk flexion and rotation as the most effective criteria for distinguishing sport classes. Following the functional assessment model described above, both studies focused either on isolated aspects (isometric strength and trunk balance) or on VoA-capacity, in relation to the player's final sport class, without considering the different stages of functional assessment in an integrated way.

In this study, we conducted an impairment based measure (Manual Muscle Testing) and a physical performance measure (trunk VoA-capacity) and analyze the complementary value of each stage to the sport class allocation (based on observed VoA-performance). In this way, we controlled the three stages of the functional model (see Figure 1). The aim of this study was to investigate how trunk and hip muscle strength contributes to VoA-capacity and, subsequently, to evaluate the effect of VoA-capacity on sport class allocation in WB. Additionally, the study aimed to compare trunk and hip muscle strength and VoA-capacity variables across different sport classes to characterize functional differences between players’ profiles.

## 2 Methods

### 2.1 Study design

This was a cross-sectional, observational study with a quantitative approach in WB players. This study was approved by the Brazilian Research Ethics Committee (CAAE: 85177424.4.0000.5404), the Senate Commission of Research Studies Ethics of the Józef Piłsudski University of Physical Education in Warsaw (SKE 01-28/2024), and the Swedish Ethical Review Authority (Dnr 2023-00990-01). All participants signed a written informed consent before participating in the study.

### 2.2 Sample

The sample consisted of 40 WB players (mean 23.3 years; Male = 34, Female = 6) from Brazil (*n* = 29) and Poland (*n* = 11), playing at a national (*n* = 26) and international level (*n* = 14). Participants had a mean height of 1.71 ± 0.11 m, a self-reported body mass of 73.8 ± 15.9 kg, and a mean training volume of 10.1 ± 6.1 h/week. Players were selected by convenience, and only those who had previously undergone an official WB classification process (national and international) were considered eligible. To align with the aim of investigating the functional basis of the current WB classification system, the inclusion criteria were: being classified in one of the main sport classes (1.0, 2.0, 3.0, 4.0, or 4.5); being 18 years of age or older; not presenting impairment in upper limbs, and being able to perform the Manual Muscle Test (MMT) assessment on a bench and VoA-capacity in their own sports wheelchair.

### 2.3 Procedures

The players’ trunk muscle strength (movements: flexion, extension, rotation (left/right), lateral flexion (left/right), and lumbar extension + hip extension) and hip muscle strength (movements: flexion, extension, adduction, and abduction) were assessed using the MMT in prone or supine positions, by three physiotherapist evaluators with extensive experience in MMT within Paralympic sports (more than five years), two of whom are certified international medical classifiers. The same three evaluators conducted all assessments, working together throughout the evaluation process to ensure methodological consistency across locations and minimize inter-rater variability. To assess trunk muscle strength, the protocol described in the Guidelines for the Trunk Test for Paracanoe athletes (5, 6) were used with adjustments based on Daniels and Worthingham's Muscle Testing protocol. This protocol uses a 0–2 scale, where 0 indicates no active movement, 1 indicates partial range of motion against gravity, and 2 indicates full range of motion against gravity and against standardized manual resistance applied by the examiner.

The hip MMT assessment followed the Daniels and Worthingham's Muscle Testing (7, 8) and Clarkson (9). This scale is from 0 to 5, where 0 represents the absence of visible or palpable muscle contraction, while 5 indicates full movement of the body segment against gravity with maximal resistance. Intermediate scores reflect progressive increases in functional capacity: 1 = visible or palpable contraction without movement; 2 = full movement with gravity eliminated; 3 = full movement against gravity without resistance; and 4 = full movement against gravity with moderate resistance. Thus, the maximum score (sum of all movements previously described: trunk = 7 movements and hip =4 movements) for the trunk is 14, the right hip is 20, and the left hip is 20. The participant characteristics are presented in Table 2.

**Table 2:** Participant characteristics with MMT presented as IQR and range for participants in different WB sport classes.

Functional aspects	Class 1.0	Class 2.0	Class 3.0	Class 4.0	Class 4.5
	Median (IQR)				
MMT of trunk	1 (0–2)	11 (8–11)	13 (11–14)	14 (14–14)	14 (14–14)
MMT of hip	0 (0–0)	2 (0–28)	25 (17–30)	39 (35–40)	40 (40–40)
Total Trunk-Hip MMT	1 (0–2)	12 (8–16)	20 (18–23)	27 (26–28)	28 (28–28)
VoA-capacity (deg)	187 (150–212)	299 (266–358)	361 (333–403)	437 (390–440)	419 (410–431)
Underlying Health Condition	Number of participants				
Spinal conditions^a^	9	6	5	1	0
Amputation	0	0	0	6	4
Others^b^	0	1	3	3	2

VoA-capacity (deg) was obtained by summing the Range of Motion (RoM) of the six movements (flexion, extension, rotation (right and left) and lateral flexion (right and left)) obtained through the IMU.

IQR, interquartile range; MMT, manual muscle test.

a Spinal cord injury, spina bifida, transverse myelitis.

b Malformation, cerebral palsy, arthrogryposis.

The players also performed maximal trunk flexion, extension, right rotation, left rotation, right lateral flexion, and left lateral flexion movements, seated in their sports wheelchairs and using all adaptive equipment they typically use for play (e.g., abdominal binder, pelvic-, legs-, and feet-straps). VoA-capacity was obtained by summing the RoMs of the six movements (flexion, extension, rotation (right and left) and lateral flexion (right and left)). According to the IWBF Classification Manual (2), VoA should be assessed under unsupported conditions, meaning that the player does not receive postural support from the ergonomic setup of the wheelchair. However, current classification practice follows the International Paralympic Committee (IPC) Classification Code (10), whereby VoA-capacity is assessed under supported conditions using the athlete's own sports wheelchair, including all straps and belts normally used during competition. Although this procedural update has not yet been incorporated into the published IWBF Classification Manual, it is currently implemented in practice. Therefore, in the present study, VoA-capacity was assessed under supported conditions. The players were instructed to sit upright in their sports wheelchairs and keep their hands overlapping on their sternum, with their elbows close to their body. Before all the moves, the players performed a test attempt. At the evaluator's command, the player performed two consecutive attempts of the same trunk movement. During these movements, the players wore an IMU (Inertial Measurement Unit) (x-IMU3, X-I/O Technologies, Bristol, UK), equipped with a tri-axial accelerometer, a tri-axial gyroscope, a tri-axial magnetometer, and an AHRS algorithm providing sensor orientation with respect to the earth reference frame, positioned on the 7th cervical vertebra (11, 12). The order of the assessment sessions (MMT and VoA-capacity) was randomized.

### 2.4 Data analysis and statistics

IMU data were collected at 100 Hz using x-IMU3 software and processed in MATLAB R2024b (The MathWorks, Natick, MA, USA). All recordings were aligned with the gravity vector and reoriented to a consistent reference frame (Z vertical, Y left, X backward) to standardize flexion–extension, lateral flexion, and axial rotation directions. Before computing trunk range of motion (RoM), the initial neutral sitting orientation recorded at the beginning of each trial was used as the reference position, and subsequent angular displacements were calculated relative to this baseline to remove individual postural offsets. A custom MATLAB script was then used to compute the RoMby identifying the maximum angles for flexion–extension, lateral flexion, and axial rotation from quaternion data. For each trial, peak angular values were extracted, and the highest value across attempts was retained for the analysis. To compute the total Trunk-Hip muscle strength (total Trunk-Hip MMT), the hip scores from both sides were first summed and normalized to the trunk scale. This normalized hip value was then added to the trunk MMT score (maximum score = 28), resulting in the following formula:

$$\mathrm{Total}\;\mathrm{Trunk}-\mathrm{Hip}\;\mathrm{MMT}=\frac{\left(right\;hip\;+\;left\;hip\right)}{40}\times 14+trunk$$

Descriptive statistics (median and interquartile range) were used to characterize the sample. The Shapiro–Wilk test was applied to assess the normality of the numeric data (VoA-capacity, total Trunk-Hip MMT). To answer how trunk and hip muscle strength contributes to VoA-capacity, a linear regression model was performed with VoA-capacity as the dependent variable and total Trunk-Hip MMT as predictor variables. Subsequently, to evaluate the effect of VoA-capacity on sport class allocation in WB, a univariate ordinal regression model was fitted. The sport class was used as the dependent variable and the VoA-capacity as the predictor variable. The sport class was obtained from the official classification procedure prior to the study. The ordinal model was chosen due to the ordinal nature of the sport class, categorized into five ordered levels (1.0, 2.0, 3.0, 4.0, and 4.5) (13). For comparisons of muscle strength (total Trunk-Hip MMT) and VoA-capacity across sport classes, the non-parametric Kruskal–Wallis test was used due to the non-normal distribution of the variables. When a significant overall effect was identified, Dunn's test with Bonferroni correction was performed for pairwise comparisons between groups.

For regressions analysis, coefficient of determination (*r*^2^), coefficient estimates (*β*), standard errors (SE), z-values and *p*-values were reported. For the Kruskal–Wallis analyses, Chi-square (*x*^2^) values, degrees of freedom (df), *p*-values, and effect sizes (*η*^2^(H)) were reported. For pairwise comparisons, adjusted *p*-values and effect sizes (*r* = Z/√N) were calculated and interpreted according to Cohen's criteria (small ≥ 0.10, medium ≥ 0.30, and large ≥ 0.50) (14). Outliers in boxplots were identified using Tukey's criterion (values < Q1−1.5 × IQR or > Q3 + 1.5 × IQR). All analyses were performed in R (version 4.4.1; R Core Team, 2024), with a significance level of *p* < 0.05.

## 3 Results

**Figure 2:**
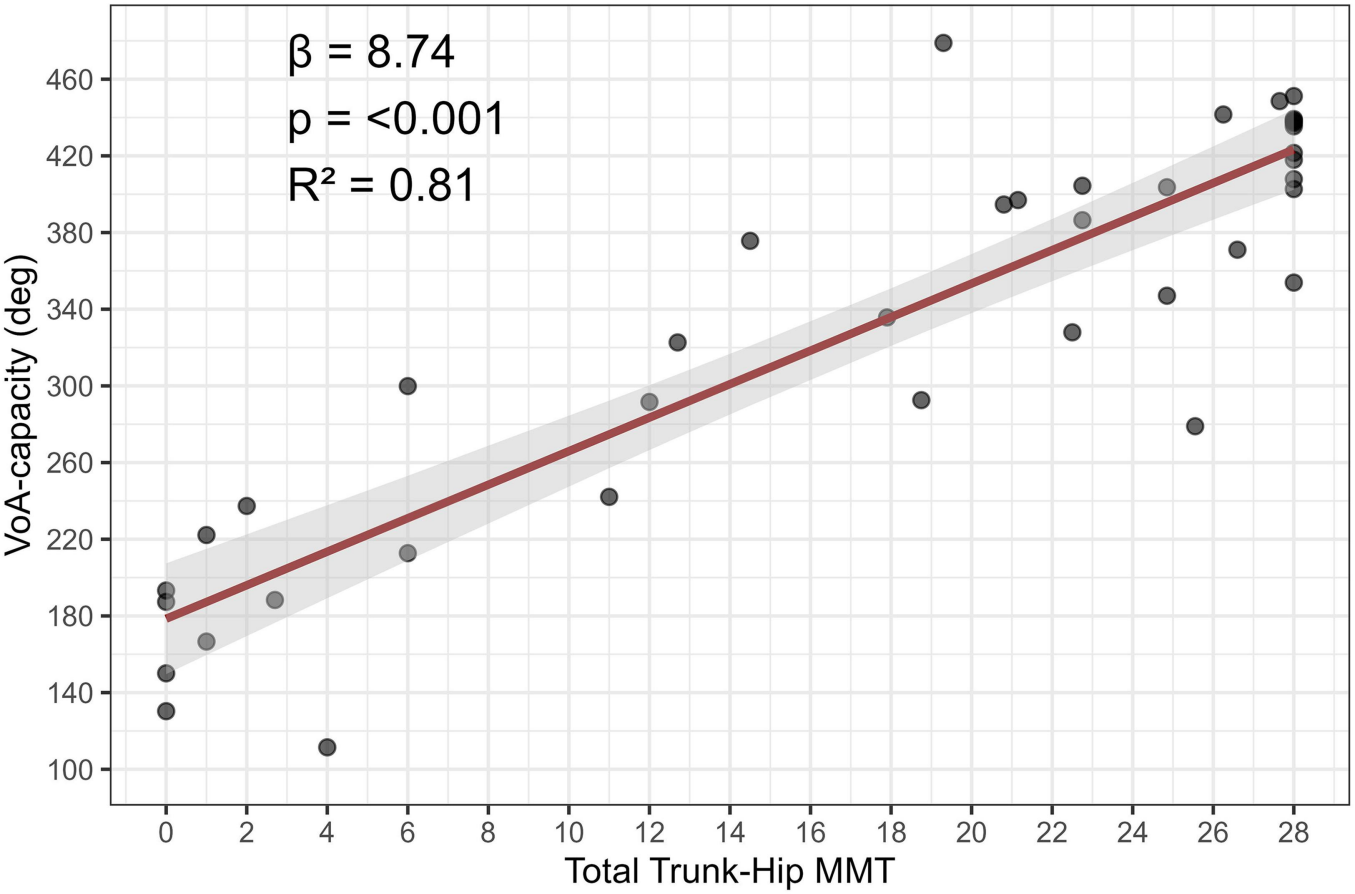
Association between total trunk-Hip MMT and VoA-capacity. *β*, coefficient estimates; *r*^2^, coefficient of determination; p, significance level; MMT, manual muscle testing; VoA, volume of action. Partial effect of total Trunk-Hip MMT on VoA-capacity (deg), with points representing observed values, line fitted by the linear model and 95% confidence band.

In the linear regression model, the total Trunk-Hip strength (total Trunk-Hip MMT) were significant predictor of VoA-capacity (*β* = 8.74, *p* = 0.001). The total Trunk-Hip MMT explained 80% of the variance in VoA-capacity (*R*^2^ = 0.80). Figure 2 illustrates the positive association between total Trunk-Hip MMT and VoA-capacity. When analyzed separately, both trunk strength (*β* = 11.64, *p* < 0.001) and hip strength (*β* = 2.18, *p* = 0.006,) were significantly associated of VoA-capacity, with the trunk explaining 38% of the variation in VoA-capacity (*R*^2^ = 0.38) and the hip explaining 18% (*R*^2^ = 0.18). In the ordinal regression model, VoA-capacity was a significant predictor of sport class. Higher VoA-capacity values were associated with higher odds of being allocated to a higher sport class (*β* = 0.027, SE = 0.0056, z = 4.84, *p* < 0.001). Also, trunk strength (*β* = 0.81, *p* = 0.020) and hip strength (*β* = 0.19, *p* = 0.002) were significantly associated with the sport class.

**Figure 3:**
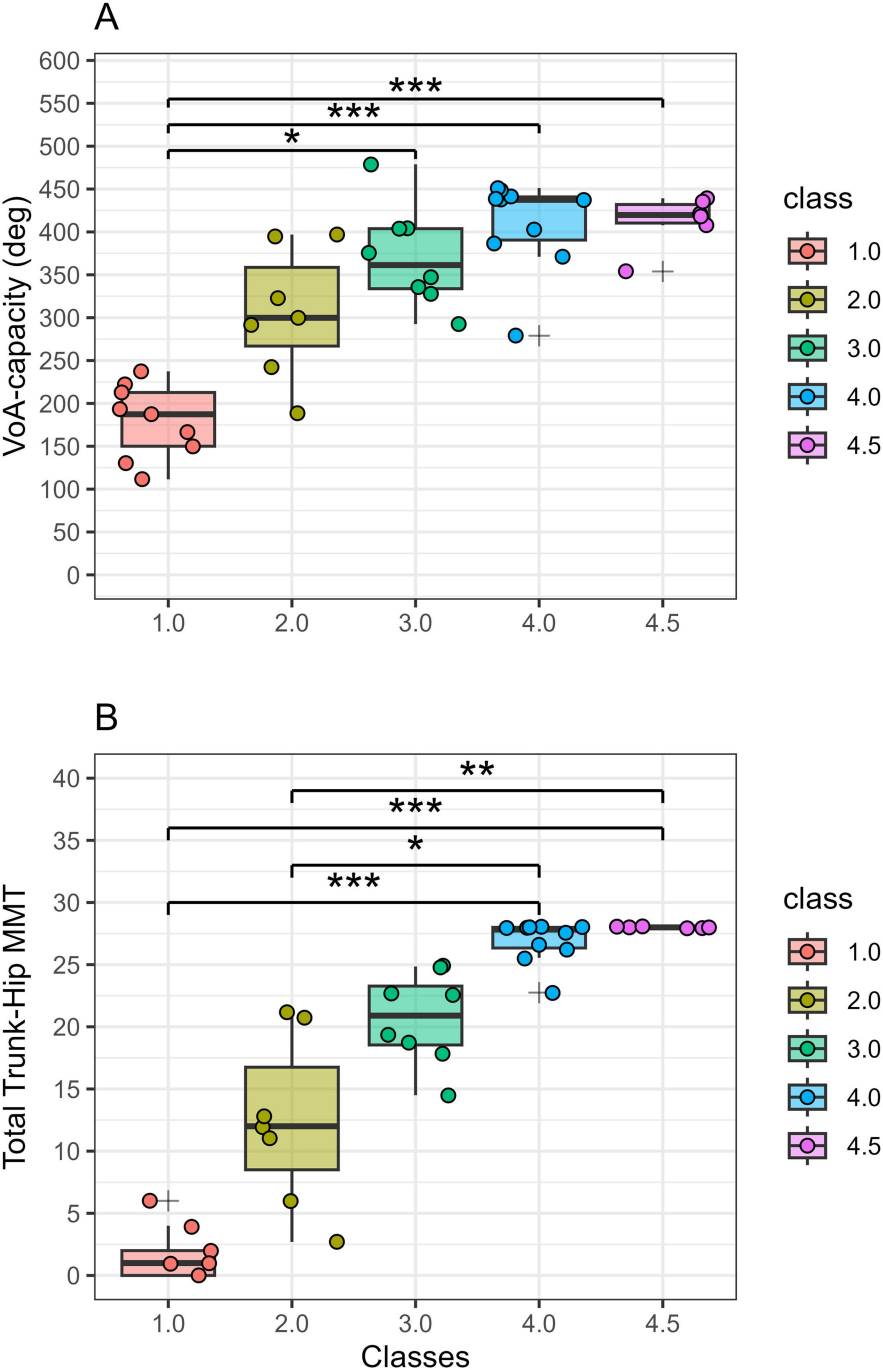
Comparison of VoA-capacity **(A)** and total trunk-Hip MMT values **(B)** between different WB sport classes. Caption: MMT, manual muscle testing; VoA, volume of action. **(A)** VoA-capacity (deg) across sport classes 1.0, 2.0, 3.0, 4.0, and 4.5. **(B)** Total Trunk-Hip MMT values across the same sport classes. Boxplots display medians, quartiles, and data distribution, with individual points representing participants. Kruskal–Wallis tests showed significant differences among sport classes for both variables. Post-hoc Dunn tests with Bonferroni correction are shown by bars and asterisks indicating significant pairwise comparisons (* = *p* < 0.05, ** = *p* < 0.01, *** = *p* < 0.001); + = outlier).

Non-parametric analyses confirmed differences between sport classes for VoA-capacity (*χ*^2^ = 26.52; df = 4; *p* < 0.001) with large effect size (*η*^2^ = 0.64) and for total Trunk-Hip MMT (*χ*^2^ = 35.29; df = 4; *p* < 0.001) with large effect size (*η*^2^ = 0.89), see Figure 3. Dunn's multiple comparisons showed differences for VoA-capacity between sport classes 1.0 and 3.0 (*p* = 0.018, *r* = 0.5), 1.0 and 4.0 (*p* < 0.001, *r* = 0.72) and 1.0 and 4.5 (*p* < 0.001, *r* = 0.62) and for total Trunk-Hip MMT between 1.0 and 4.0 (*p* < 0.001, *r* = 0.76), 2.0 and 4.0 (*p* = 0.029, *r* = 0.47), 1.0 and 4.5 (*p* < 0.001, *r* = 0.77) and 2.0 and 4.5 (*p* = 0.004, *r* = 0.52).

## 4 Discussion

This study investigated the initial stage of the WB functional classification process, examining the association between trunk and hip muscle strength and VoA-capacity, and the association between VoA-capacity and sport class allocation in WB (see Figure 1). We also compared Trunk-Hip muscle strength (total Trunk-Hip MMT) and VoA-capacity across sport classes to better understand sport class profiles. The results showed a strong association between trunk and hip muscle strength and VoA-capacity, with total Trunk-Hip MMT explaining 80% of the variance in VoA-capacity. Furthermore, VoA-capacity showed a significant association with sport class, indicating that players with higher trunk functional capacity are more likely to be allocated to higher sport classes. Comparisons between sport classes reinforced this pattern: both VoA-capacity and total Trunk-Hip muscle strength clearly differentiated lower from higher sport classes.

Isolated trunk and hip muscle strength measures define the limits of VoA by reflecting the type and level of impairment and influencing functional movement range, with variations in strength producing distinct VoA patterns that characterize each sport class. In this study, trunk and hip muscle strength together explained 80% of the variance in VoA-capacity, with particular emphasis on the trunk, which alone explains 38% of this variance. This result is linked to the sample composition, largely consisting of individuals with spinal conditions that affect voluntary muscle contraction (15–17). Although VoA-capacity depends on multiple functions, trunk and hip strength proved to be a strong predictor, aligning with the WB classification premise that emphasizes trunk and pelvic stability as primary determinants (2). This central role of the trunk is also evident in wheelchair sports such as rugby and tennis (18), where trunk strength and coordination are essential for the controlled execution of movements (18–23). Consequently, players across sport classes exhibit distinct trunk muscle strength levels and activation patterns, with a progressive increase observed in higher sport classes (22, 23).

Another important finding was that VoA-capacity had a significant effect on players’ sport class, quantitatively confirming what is qualitatively assessed during the classification process, as VoA-capacity reflects the same functional distinctions used to determine sport class allocation. While different sport classes exhibit distinct VoA patterns due to limitations in underlying functional components, VoA is also influenced by factors such as movement technique, time since injury, compensatory actions, and wheelchair ergonomics (24, 25). In this study, all assessments were performed with the athletes seated and strapped in their own sports wheelchairs. This configuration is known to influence trunk mechanics by enhancing stability in athletes from lower sport classes while potentially restricting trunk movement in athletes from higher sport classes (26). Consequently, the contribution of trunk and hip muscle strength to VoA-capacity may differ according to the strapping configuration. Additionally, compensatory muscle recruitment may influence VoA-capacity. Players with mild to moderate impairments may increase activation of the rectus abdominis to enhance anterior trunk stability compared to individuals without impairment (27). In contrast, players with more severe impairments rely predominantly on muscles such as the latissimus dorsi and anterior deltoid to compensate for limited trunk stability (27). Coordination also plays a role, as trunk balance and postural control are more limited in conditions such as ataxia or athetosis due to altered force, rhythm, and precision (44–46), however, no players in this sample presented coordination impairments.

Distinguishing between VoA-capacity and VoA-performance is essential, as they are assessed under different conditions. VoA-capacity refers to trunk movement assessed under controlled conditions, isolating specific components to evaluate maximal voluntary function, capturing the ‘function of the part’ without interference from game demands (1). In contrast, VoA-performance reflects the integration of multiple tasks in dynamic, game-like contexts, where trunk control interacts with wheelchair and ball handling, technical skills, and tactical decision-making, while also being influenced by cognitive and psychological demands (28, 29). Importantly, sport class allocation is based on VoA-performance observed during sport-specific actions, reflecting a ‘big picture’ assessment of how players use their available trunk function in real game contexts (1). For example, when involved in a collision, a 1.0-player typically loses balance, whereas a 4.0-player can maintain stability. VoA-performance also explains differences in wheeled mobility performance (WMP), which emerges from interactions between the player, wheelchair, and competitive environment. Players with higher VoA-performance (higher sport classes) tend to exhibit better speed, acceleration and mobility capabilities due to better trunk alignment, more effective force transmission, and greater stability during accelerations, decelerations, and collisions (30–38). However, among high-level players, there is no scientific consensus that higher-sport class athletes are systematically faster than lower-sport class players, as factors such as propulsion technique, experience, training, and wheelchair configuration also strongly influence wheeled mobility performance (39, 40). Thus, classification influences skills that require trunk stability (such as ball handling skills: passing/catching, shooting/rebounding and dribbling) more than speed and acceleration itself (41).

When comparing total Trunk-Hip muscle strength with VoA-capacity across sport classes, both variables showed significant differences but with distinct patterns reflecting their nature. Total Trunk-Hip strength primarily distinguished lower (1.0–2.0) from higher sport classes (4.0–4.5), whereas VoA-capacity discriminated sport class 1.0 from sport classes 3.0 (middle-sport class), 4.0, and 4.5 (higher-sport class). The progressive increase in Trunk-Hip strength across sport classes is consistent with the findings of Santos et al. (3), who reported greater trunk isometric strength and stability limits in athletes from higher sport classes. These sport class-discrimination patterns likely reflect the sample characteristics of this study: lower sport classes predominantly included players with spinal cord conditions affecting muscle strength, whereas higher sport classes consisted mainly of athletes with lower-limb amputations or conditions not directly impairing strength. In contrast, VoA-capacity represents a functional assessment that integrates trunk strength with postural control, balance, coordination, and movement strategy during standardized sport-specific tasks, consistent with the evidence-based classification framework proposed by the IPC, in which sport classes are determined by the impact of the eligible impairment on sport-specific performance rather than by isolated impairment measures (10). Although both measures reflected the overall functional profiles of the sport classes, neither discriminated adjacent sport classes. This lack of sport class discrimination is partly explained by the use of composite scores that sum the three movement planes (VoA-capacity). Methodological differences also contribute to the lack of discrimination between adjacent sport classes: total Trunk-Hip strength is assessed without external stabilization, making impairments more evident, whereas VoA-capacity is measured in a supported, sport-specific context in the wheelchair, where backrest, seat configuration, and strapping provide stability and allow greater trunk RoM, especially among lower-sport class players (4). Ultimately, sport class allocation is based on VoA-performance observed in dynamic game situations, where multiple interacting factors come into play, and subtle distinctions between adjacent sport classes emerge only through this more complex, real-game assessment.

Trunk-Hip muscle strength and VoA-capacity offer complementary insights on a player's functional profile. MMT characterizes isolated muscle strength, helping explain stability, reach, and compensatory strategies, whereas VoA-capacity reflects how this strength is expressed during functional testing under controlled conditions. Together, they help anticipate VoA-performance and support interpretation during classification, especially in challenging cases by clarifying whether observed limitations, such as loss of balance or reduced trunk excursion, align with the player's functional profile or suggest alternative mechanisms. In the current IWBF assessment process, MMT contributes to determining MIC, VoA-capacity informs the initial sport class, and the final sport class is based on VoA-performance (2). Integrating muscle strength and VoA-capacity improves the understanding of VoA-performance in more challenging cases.

This study contributes to understanding sport class allocation in WB but has limitations that should be considered. The sample was predominantly male, which limits the generalizability of the findings to female players. The use of MMT, although common, is qualitative and subject to inter- and intra-rater variability (42), with reduced sensitivity at higher strength levels (grades 3–5), which may explain the lack of differences between higher sport classes. Additionally, allowing players to use their own wheelchair configurations introduced variability in ergonomic factors that influence stability, reach, and mobility (43). Future research should examine the relationship between VoA-capacity and VoA-performance, determine how VoA-performance informs sport class allocation by identifying key observable behaviors (e.g., loss of balance, trunk excursion, recovery strategies), and explore its links to sport-specific activities such as dribbling, passing, shooting, and wheeled mobility performance. Furthermore, the assessment of VoA-performance in ecologically valid, competition-like environments, such as small-sided games, may provide valuable insights into trunk function under dynamic and multitask conditions, better reflecting the game-observation context used in the classification process.

## 5 Conclusion

Functional assessment in WB classification is a complex, multifactorial process. This study offers preliminary evidence regarding functional relationships relevant to WB classification. The results indicate associations between trunk and hip muscle strength and VoA-capacity, suggesting how isolated muscle strength may relate to players’ ability to perform dynamic seated movements. VoA-capacity also showed anassociation with sport class, which may reflect functional distinctions considered during classification. The observed associations highlight the potential value of considering trunk and hip muscle strength as complementary information in challenging classification situations, even though muscle strength is not part of the formal criteria for class allocation. Future investigations integrating VoA performance will therefore be essential to fully evaluate the classification assessment model. Overall, this study contributes initial insights into the relationships among muscle function, functional measures, performance, and sport class allocation.

## Data Availability

The raw data supporting the conclusions of this article will be made available by the authors, without undue reservation.
